# Henipaviruses at the threshold: preparedness in the era of recurrent spillover

**DOI:** 10.1080/22221751.2026.2667556

**Published:** 2026-06-09

**Authors:** Xinyu Wang, Wenhong Zhang

**Affiliations:** Department of Infectious Diseases, Huashan Hospital, Fudan University, Shanghai, People’s Republic of China

**Keywords:** Henipavirus, Nipah virus, Hendra virus, zoonotic spillover, One Health, outbreak preparedness, vaccine evaluation, emerging infectious diseases

## Abstract

Henipaviruses remain high-consequence zoonotic pathogens characterized by recurrent spillover, severe disease, and limited but consequential human-to-human transmission. In this commentary, we frame henipaviruses as “threshold pathogens”: agents repeatedly approaching, but not yet consistently crossing, the boundary into sustained community transmission. Expanding viral diversity, widening ecological interfaces, and episodic outbreaks challenge conventional preparedness models, particularly for surveillance, countermeasure evaluation, and regulatory decision-making. We argue that future preparedness should move beyond outbreak counting towards integrated One Health surveillance, adaptive evidence-generation pathways, and trial-ready countermeasure strategies suited to pathogens with irregular emergence and high public health consequences.

Since its first recognized outbreak in Malaysia in 1998–1999, Nipah virus has caused recurrent spillover events across South and Southeast Asia for more than two decades [1]. Although outbreaks have generally remained limited in size, case fatality rates have frequently exceeded 60% and in some outbreaks approached 100%, underscoring its capacity for severe human disease. Hendra virus, within the same genus, has caused sporadic human infections in Australia [2]. Together, these patterns illustrate that highly pathogenic henipaviruses are not confined to a single ecological setting. Yet their emergence has been episodic rather than sustained, complicating conventional preparedness models. The question is no longer whether henipaviruses can spill over, but how to interpret a pattern of recurrent, geographically shifting emergence in the absence of continuous transmission. Here, we argue that henipaviruses can be understood as “threshold pathogens”: agents capable of repeated zoonotic spillover and severe human disease, yet not consistently achieving sustained community transmission. This framing provides a conceptual basis for reinterpreting preparedness challenges, shifting attention from outbreak counting alone to the interaction between ecological exposure, transmission constraint, and preparedness capacity.

Reconsideration of this pattern has become increasingly necessary as the recognized diversity of henipa-related viruses expands. Taxonomic revisions have refined the classification of henipa-related viruses under the ICTV 2024 framework [3]. In parallel, intensified wildlife surveillance has revealed a broader evolutionary landscape and geographic spread than previously recognized [4]. The significance of this expansion extends beyond classification. It reflects a widening evolutionary space within which zoonotic potential may arise, even if most identified lineages have not been associated with human disease. Importantly, broader viral discovery and wider geographic detection should not be interpreted as evidence of confirmed human pathogenicity.

Geographic signals reinforce this concern. While recognized human Nipah virus disease has been concentrated in South Asia, and Hendra virus infections in Australia, surveillance has identified henipa-related viruses across multiple continents. Langya virus, detected in febrile patients in China and linked epidemiologically to shrews, demonstrates that human spillover is not restricted to bat-associated viruses [5]. Recognition of henipa-related viruses in wildlife has expanded steadily over the past decade (Table 1). A nosocomial cluster reported in early 2026, involving two laboratory-confirmed cases among healthcare workers at a private hospital in West Bengal, demonstrated how limited spillover events can test infection control resilience even without sustained community transmission [6]. Collectively, these findings suggest that the apparent geographic concentration of recognized disease may reflect surveillance intensity rather than true restriction of viral circulation, and that case maps may substantially underestimate the wider ecological footprint of henipa-related viruses.

**Table 1.** Chronological expansion of recognized henipa-related viruses in wildlife.

Virus	Genus (ICTV 2024 framework)	Primary reservoir host	Region first reported	Year first described*	Human infection reported?
Saltwater Creek virus (SGV)	Henipa-related lineage	Bats	Australia	2012	No
Mojiang virus	Parahenipavirus	Rodents	China	2012	No confirmed
Angavokely virus (AngV)	Henipa-related lineage	Bats	Madagascar	2018	No
Langya virus (LayV)	Parahenipavirus	Shrews	China	2022	Yes
Camp Hill virus	Parahenipavirus	Shrews	USA	2025	No

*Year refers to first description in the peer-reviewed scientific literature. Classical henipaviruses (Nipah virus and Hendra virus) are not shown. Genus assignments follow the ICTV 2024 taxonomy framework.

Importantly, expanding diversity does not equate to imminent adaptation for efficient human transmission. In this commentary, the “threshold” refers specifically to the boundary between recurrent zoonotic spillover with limited transmission chains and sustained human-to-human transmission capable of supporting community spread. Longitudinal investigations in Bangladesh and India indicate repeated spillover from bat reservoirs with limited chains of person-to-person transmission [7]. Current genomic analyses do not demonstrate sustained adaptive changes associated with enhanced human transmissibility, nor evidence that increasing spillover reflects accelerated viral evolution rather than ecological amplification [8]. In this respect, henipaviruses remain pathogens of high consequence but constrained transmissibility – precisely the kind of threat for which preparedness frameworks are often least well calibrated.

However, ecological conditions under which spillover occurs are becoming more permissive. Agricultural intensification, deforestation, wildlife habitat fragmentation, expanding livestock interfaces and increasing human encroachment into bat and small-mammal habitats alter contact patterns between reservoirs and humans [9]. Climate variability may further influence reservoir distribution and viral shedding dynamics. Longitudinal field investigations have demonstrated that synchronous viral shedding in bat populations can coincide with peak periods of spillover, reinforcing the importance of ecological timing in outbreak emergence [10]. These structural pressures do not guarantee adaptation, but they increase opportunities for cross-species transmission and amplification events. The resulting risk landscape is therefore characterized not by inevitability, but by repeated exposure of the human population to zoonotic interfaces.

This dynamic creates a preparedness dilemma. The dilemma is not simply that countermeasures are lacking, but that the epidemiological pattern of recurrent, low-incidence emergence is structurally misaligned with conventional evidence-generation pathways. Nipah virus is consistently listed among priority pathogens for research and development, yet vaccine efficacy evaluation remains constrained by the irregular and geographically scattered nature of outbreaks. Traditional phase III efficacy trials require predictable incidence and sufficient case accrual, conditions rarely met in the context of episodic spillover [11]. Even when candidate vaccines or monoclonal antibodies demonstrate immunogenicity and safety in early-phase studies, generating definitive efficacy data becomes logistically and ethically complex [12].

Ring vaccination strategies have been proposed as a potential solution in settings where large-scale randomized trials are infeasible. The successful application of ring vaccination during the West African Ebola epidemic provides a conceptual precedent [13]. In principle, such designs may allow outbreak-embedded evaluation while simultaneously contributing to containment. In practice, however, several challenges arise in the henipavirus context. Outbreaks are often small and geographically dispersed, limiting statistical power. The absence of established correlates of protection complicates interpretation of immunogenicity endpoints. Rapid identification of index cases and contacts requires surveillance systems capable of real-time response. Ethical and regulatory approval processes must function within compressed timeframes. Under these conditions, ring vaccination may represent a theoretically attractive framework, but its feasibility as a robust efficacy pathway remains uncertain. Its significance may lie less in offering a ready solution than in exposing how future preparedness will depend on trial-ready protocols, adaptive regulation, and outbreak-responsive evidence generation.

The challenge, therefore, is not solely scientific but institutional. Preparedness for pathogens characterized by high lethality, recurrent spillover and uncertain transmissibility requires governance structures capable of operating in the space between emergency response and routine public health practice. Regulatory pathways must accommodate evidence generated under outbreak-embedded designs. Surveillance systems must integrate wildlife, veterinary and human health data within a One Health framework. Manufacturing and stockpiling strategies must be calibrated for pathogens that may never achieve sustained human transmission, yet carry disproportionate consequence when spillover occurs.

Henipaviruses thus occupy a distinctive position in the global risk spectrum. They have demonstrated capacity for severe disease and limited human-to-human transmission, but have not crossed the threshold into sustained community spread. At the same time, the recognized ecological and geographic footprint of henipa-related viruses continues to expand. The absence of sustained transmission should not be misinterpreted as stability. Rather, it reflects a balance between biological constraints and ecological exposure that may shift over time.

In this sense, henipaviruses represent a test case for preparedness paradigms in an era of accelerating environmental change and expanding pathogen discovery. The critical question is not whether the next outbreak will be larger than the last, but whether current systems are designed to detect, evaluate and contain pathogens that repeatedly approach – yet have not definitively crossed – the boundary of sustained human adaptation. Building such systems requires investment before crisis, coordination across disciplines, and regulatory flexibility matched to episodic emergence. At a minimum, this requires three shifts in preparedness thinking: integrating wildlife, veterinary and human surveillance more effectively; developing adaptive regulatory pathways for outbreak-based evidence generation; and investing in countermeasure platforms that can be evaluated under conditions of episodic emergence. Whether henipaviruses remain constrained zoonotic threats or evolve into broader public health challenges will depend not only on viral biology, but on how effectively global health institutions respond to signals at the threshold.
